# Characterization of the Merkel Cell Carcinoma miRNome

**DOI:** 10.1155/2014/289548

**Published:** 2014-02-03

**Authors:** Matthew S. Ning, Annette S. Kim, Nripesh Prasad, Shawn E. Levy, Huiqiu Zhang, Thomas Andl

**Affiliations:** ^1^Division of Dermatology, Department of Medicine, Vanderbilt University Medical Center, Medical Center North, Room A2310A, 1161 21st Avenue South, Nashville, TN 37232-2600, USA; ^2^Department of Pathology, Microbiology, and Immunology, Vanderbilt University Medical Center, Nashville, TN 37232-2600, USA; ^3^Department of Biological Sciences, University of Alabama in Huntsville, Huntsville, AL 35805, USA; ^4^HudsonAlpha Institute for Biotechnology, Huntsville, AL 35806, USA; ^5^OrioR Lab, LLC, Rockville, MD 20852, USA

## Abstract

MicroRNAs have been implicated in various skin cancers, including melanoma, squamous cell carcinoma, and basal cell carcinoma; however, the expression of microRNAs and their role in Merkel cell carcinoma (MCC) have yet to be explored in depth. To identify microRNAs specific to MCC (MCC-miRs), next-generation sequencing (NGS) of small RNA libraries was performed on different tissue samples including MCCs, other cutaneous tumors, and normal skin. Comparison of the profiles identified several microRNAs upregulated and downregulated in MCC. For validation, their expression was measured via qRT-PCR in a larger group of MCC and in a comparison group of non-MCC cutaneous tumors and normal skin. Eight microRNAs were upregulated in MCC: miR-502-3p, miR-9, miR-7, miR-340, miR-182, miR-190b, miR-873, and miR-183. Three microRNAs were downregulated: miR-3170, miR-125b, and miR-374c. Many of these MCC-miRs, the miR-183/182/96a cistron in particular, have connections to tumorigenic pathways implicated in MCC pathogenesis. *In situ* hybridization confirmed that the highly expressed MCC-miR, miR-182, is localized within tumor cells. Furthermore, NGS and qRT-PCR reveal that several of these MCC-miRs are highly expressed in the patient-derived MCC cell line, MS-1. These data indicate that we have identified a set of MCC-miRs with important implications for MCC research.

## 1. Introduction

Merkel cell carcinoma (MCC) is a primary neuroendocrine carcinoma of the skin of uncertain origin. Although not as prevalent as other skin cancers, MCC is aggressive and has a high mortality rate, with an overall five-year survival of sixty percent [1]. Median survival for patients with and without regional lymph node involvement at presentation is thirteen and forty months, respectively [2], and up to fifty percent of patients eventually develop systemic disease with metastases to liver, bone, and brain [3].

Unfortunately, while the incidence of MCC increases, our knowledge of these tumors remains limited. Several factors are implicated in its pathogenesis, including UV radiation exposure, an associated polyomavirus (MCPyV), and immunosuppression [4, 5], but the lack of effective treatment options available for MCC reflects our limited knowledge. In an attempt to expand our understanding, we focus our attention on microRNAs, small single-stranded RNA molecules that participate in the negative regulation of gene expression.

With average lengths of roughly twenty-two nucleotides, these molecules serve as guides of the RNA-induced silencing complex (RISC). MicroRNAs regulate the expression of genes by binding to partially complementary target sites in mRNA transcripts and inhibiting their translation [6]. A wealth of data has accumulated in recent years implicating microRNAs as significant modulators of gene expression, and through their unique role in posttranscriptional regulation, they can function as important regulators of tumor growth and metastasis.

The study of microRNAs holds much promise for improving the diagnosis and treatment of cancer. Recent progress in our understanding of the role of microRNAs in disease has excited oncology. An effective antiviral therapy was recently developed based on microRNA biology, and microRNAs are promising new tools and targets in cancer research [7].

Indeed, microRNAs have been demonstrated to play significant roles in the pathogenesis of other skin cancers, such as squamous cell carcinoma (SCC) and melanoma [6]. They have also been implicated in basal cell carcinoma (BCC) and may even be employed in the accurate identification of cancer subtype, as demonstrated for melanoma and cutaneous T-cell lymphoma (CTCL) [6]. However, despite the volume of literature on microRNAs in skin cancer, little is yet known about the role that these molecules play in MCC. Thus, we set out to close this knowledge gap and define the microRNAs involved in MCC biology.

## 2. Methods

### 2.1. Samples

Frozen tissue samples of various skin cancers (MCC, melanoma, SCC, and BCC), normal skin, and normal lymph node were obtained from the Cooperative Human Tissue Network (CHTN) and stored at −80°C. Formalin-fixed paraffin-embedded (FFPE) tissue samples of various skin cancers (MCC, melanoma, SCC, and BCC) and normal skin were obtained from Vanderbilt Pathology and Dermatopathology (Nashville, TN). Clinical information corresponding to the FFPE MCC samples is provided (Table 1).

### 2.2. Cell Lines

All cell lines were cultured in DMEM with 10% FCS and Pen-Strep.

### 2.3. RNA Isolation

Total RNA, including microRNAs, was isolated from frozen tissue and cell culture with the miRNeasy Mini Kit and from FFPE tissue with the miRNeasy FFPE Kit (Qiagen; Hilden, Germany), according to the manufacturer's protocols. The concentration and integrity of the extracted total RNA were estimated by Qubit 2.0 Fluorometer (Invitrogen, Carlsbad, CA, USA) and Agilent 2100 Bioanalyzer (Applied Biosystems, Carlsbad, CA, USA), respectively. RNA samples with a RNA Integrity Number (RIN) value of at least 7.0 or higher was used for further processing.

### 2.4. Small RNA (miRNA) Library Preparation and Sequencing

Approximately 1 *μ*g of total RNA from each sample was taken into small RNA library preparation protocol using NEBNext Small RNA Library Prep Set for Illumina (New England BioLabs Inc., Ipswich, MA, USA) according to manufacturer's protocol. Briefly, 3′ adapters were ligated to total input RNA followed by hybridization of multiplex SR RT primers and ligation of multiplex 5′ SR adapters. Reverse transcription (RT) was done using SuperScript III RT (Life Technologies, Grand Island, NY, USA) for 1 hour at 50°C. Immediately after RT reaction, PCR amplification was performed for 15 cycles using LongAmp Taq 2X master mix. Illumina indexed primers were added to uniquely barcode each sample. Post-PCR material was purified using QIAquick PCR purification kit (Qiagen Inc., Valencia, CA, USA). Post-PCR yield and concentration of the prepared libraries were assessed using Qubit 2.0 Fluorometer and DNA 1000 chip on Agilent 2100 Bioanalyzer. Size selection of small RNA with a target size range of 140 bp was done by running samples on a 6% PAGE gel for 1 hour at 120 V. Accurate quantification for sequencing applications was performed using the qPCR-based KAPA Biosystems Library Quantification kit. Each library was diluted to a final concentration of 12.5 nM and pooled in equimolar ratios prior to clustering. Cluster generation was carried out on a cBot v1.4.36.0 using Illumina's Truseq Single Read (SR) Cluster Kit v3.0. Single End (SE) sequencing was performed to generate at least 15 million reads per sample on an Illumina HiSeq2000, running HiSeq Control Software (HCS) v1.5.15.1, using a 50-cycle TruSeq SBS HS v3 reagent kit. The clustered flowcells were sequenced for 56 cycles, consisting of a 50-cycle read, followed by a 6-cycle index read. Image analysis and base calling was performed using the standard Illumina Pipeline consisting of Real time Analysis (RTA) version v1.13 and demultiplexed using bcl2fastq converter with default settings.

### 2.5. Processing of Small RNA-Seq Reads

At least 15 million, 50 bp, SE reads were generated from each sample. Further downstream analysis of the sequenced reads from each sample was performed as per our unique in-house pipeline. Briefly, quality control checks on raw sequence data from each sample will be performed using FastQC (Babraham Bioinformatics, London, UK). Raw reads were then imported on a commercial data analysis platform CLCbio (CLCbio, MA, USA). Adapter trimming was done to remove ligated adapter from 3′ end of the sequenced reads with only one mismatch allowed; poorly aligned 3′ ends were also trimmed. Sequences shorter than 15 nucleotides length were excluded from further analysis. Trimmed Reads were then used to extract and count the small RNA which were then annotated with microRNAs in miRBase release 18 database. Samples were grouped as per their types identifiers and quantification of miRNA abundance was done. Differential expression of miRNA was calculated on the basis of their fold change (default cut-off ≥±2.0) between mapped counts observed between individual groups.

### 2.6. Quantitative Real-Time Reverse Transcription Polymerase Chain Reaction (qRT-PCR)

To validate the NGS data, the expression of microRNAs was analyzed via quantitative real-time reverse transcription polymerase chain reaction (qRT-PCR) with the Rotor-Gene SYBR Green PCR Kit, miScript Primer Assays, and miScript Universal Primer, following reverse transcription of total RNA with the miScript II RT Kit (Qiagen; Hilden, Germany).

The qRT-PCR analysis was performed in technical replicates according to the manufacturer's instructions using the Rotor-Gene SYBR Green PCR Master Mix (Qiagen; Hilden, Germany). The packaged operating software was utilized for instrument control, data acquisition, and raw data analysis. The plates were run in relative quantification (ΔΔ*C*_*t*_) mode with triplicate measurements.

Amplification curves were analyzed using the packaged operating software, and assays were inspected for distinct melting curves. In addition, only assays detected with *C*_*t*_ < 35 were included in the data analysis. To calculate the relative expression levels of target microRNAs, the ΔΔ*C*_*t*_ algorithm method was utilized. miR-423-3p and miR-423-5p were stably expressed across all samples, thus the average of their *C*_*t*_s in each sample was used as the normalization factor. Assays were calibrated to the same normal skin sample.

### 2.7. *In Situ* Hybridization (ISH)

*In situ* detection of miR-182 was carried out using the AccuRISH service of OrioR Lab, LLC (Rockville, MD). An RNAse-free tissue sectioning environment was created by treating the microtome, blades, water bath, ice bucket, forceps, and slide tray with RNaseZap, followed by RNase-free water. Each 5-*μ*m tissue section was dewaxed, rehydrated, and demasked, and protease treatment was optimized for each tissue block with GAPDH probe and differing concentrations of Protease K. After treatment, tumor and normal tissue sections were stained side-by-side using an anti-miR-182 oligonucleotide probe of same concentration with same hybridization and stringent washing temperature of 55–60°C. The final color development for all sections of each batch was terminated together after ninety minutes. All pictures were taken using an Olympus DP70 digital camera with the same setting.

## 3. Results

### 3.1. Differential MicroRNA Expression Based on High-Throughput Sequencing Data

Sequencing of small RNA libraries was performed for the following frozen tissue samples: three MCCs, one melanoma, one SCC, one BCC, and one normal skin. The MCC and melanoma were lymph node metastases, while the SCC and BCC were primary cutaneous lesions. Comparison of the sequencing profiles identified several microRNAs upregulated and downregulated in MCC versus other tissues (Table 2).

### 3.2. Confirmation of MCC-miRs via qRT-PCR

**Figure 1:**
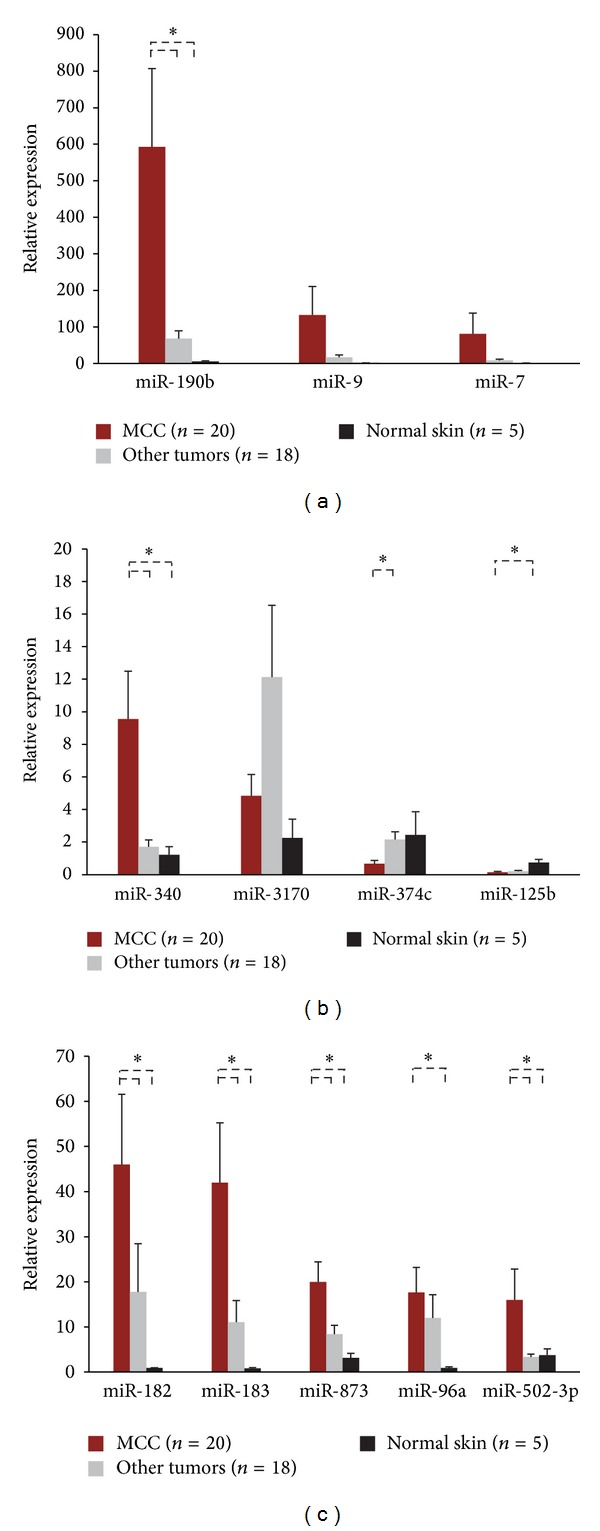
Validation of MCC-miRs via qRT-PCR. Eight microRNAs were confirmed to be upregulated in MCC versus other tumors and normal skin: miR-190b, miR-9, miR-7, miR-182, miR-183, miR-873, miR-502-3p, and miR-340. The tumor group consists of melanoma (*n* = 5), SCC (*n* = 6), and BCC (*n* = 7). Error bars refer to SEM. *Welch's *t*-test: *P* < 0.05.

To validate the next generation sequencing (NGS) data, several microRNAs were evaluated via qRT-PCR in larger cohorts of FFPE tissue samples. The MCC cohort consisted of a mixture of primary cutaneous lesions and metastases (Table 1). The tumor group consisted of primary cutaneous lesions of melanoma, SCC, and BCC (Figure 1). The qRT-PCR results confirmed the upregulation (≥2-fold) of eight microRNAs in MCC: miR-502-3p, miR-9, miR-7, miR-340, miR-182, miR-190b, miR-873, and miR-183. In addition, three microRNAs were found to be downregulated (≥2-fold) in MCC: miR-3170, miR-125b, and miR-374c. The data also identified the slight upregulation (≥1.5-fold) of miR-96a, another member of the miR-183/96/182 cluster.

**Figure 2:**
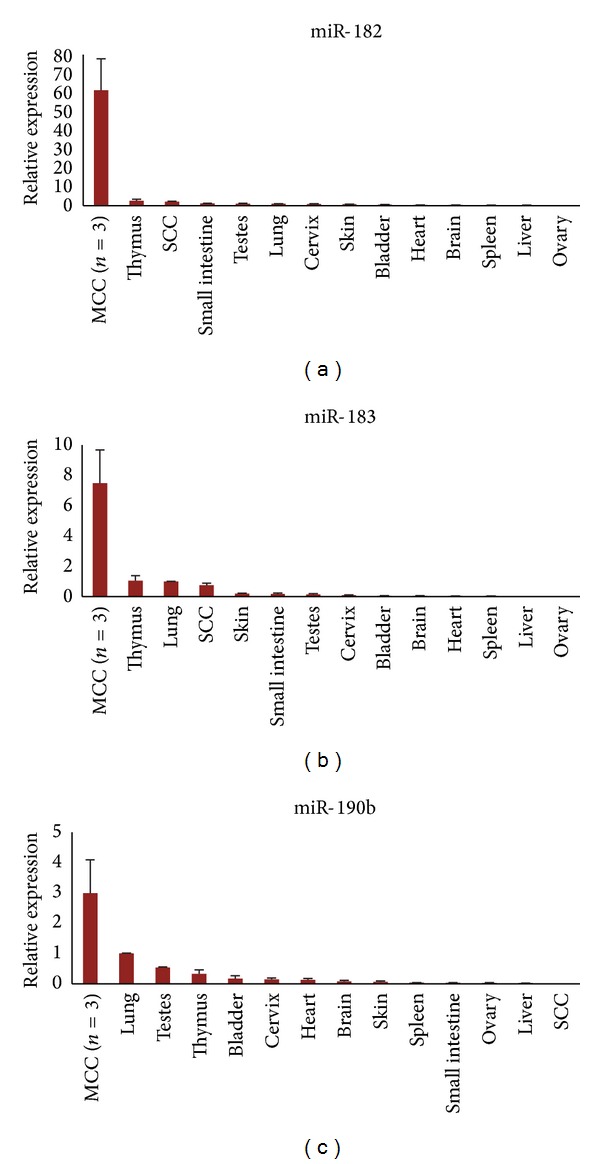
MCC-miRs are specific for MCC. Three MCC-miRs were confirmed via qRT-PCR to be upregulated in frozen MCC samples versus a human tissue panel consisting of twelve different body organs: miR-182, miR-183, and miR-190b. Error bars refer to SEM.

To assess whether these microRNAs are specific tumor markers for MCC, the expression of each of the eight MCC-miRs was evaluated via qRT-PCR in several frozen MCC lymph node metastases and compared to a human tissue panel consisting of twelve different organs (Figure 2). Three of the MCC-miRs demonstrated higher expression (≥2-fold) in the MCC cohort versus all other organs: miR-183, miR-182, and miR-190b.

### 3.3. MCC-miRs Are Highly Expressed in the MCC Cell Line, MS-1

**Figure 3:**
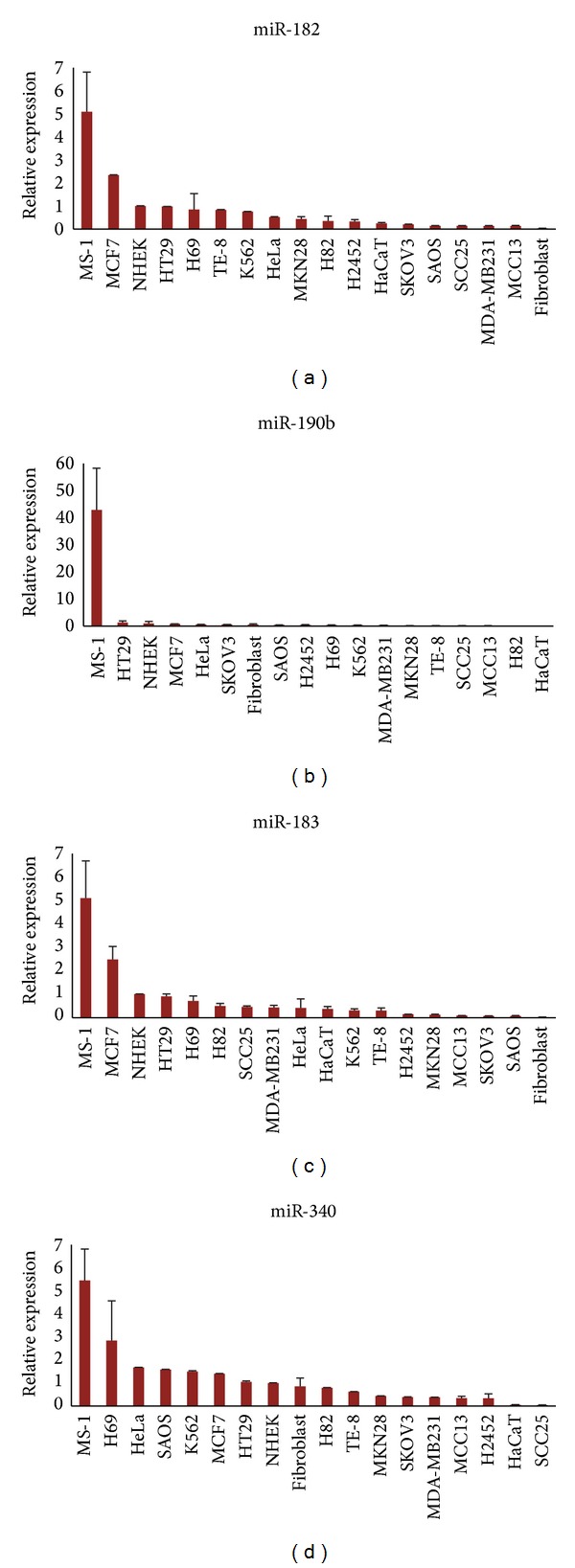
MCC-miRs are highly expressed in MS-1. Four MCC-miRs were confirmed via qRT-PCR to be upregulated in the MCC cell line, MS-1, versus sixteen other non-MCC cell lines: miR-182, miR-183, miR-190b, and miR-340. Error bars refer to SEM.

To assess the potential of these findings for future functional studies, NGS of small RNA libraries was performed for the patient derived MCPyV-positive cell line, MS-1 (Table 2). Several of the same MCC-miRs found to be upregulated in the tissue samples also demonstrated high absolute expression values in MS-1. Thus, the expression of each of the MCC-miRs was evaluated via qRT-PCR in MS-1 and compared to that of various non-MCC cell lines (Figure 3). The following MCC-miRs were confirmed to be elevated (≥2-fold) in MS-1 versus a set of sixteen non-MCC cell lines: miR-183, miR-182, miR-340, and miR-190b—interestingly, the same microRNAs were found to be upregulated in MCC versus the tissue panel. Together, these data indicate that we have identified a set of high quality MCC-miRs.

The expression of each of the four microRNAs was also evaluated via qRT-PCR in the MCPyV-negative cell line, MCC13, but with different results (Figure 3). In contrast to MS-1, the four microRNAs, miR-183, miR-182, miR-340, and miR190b demonstrated low expression levels in MCC13. The levels of these microRNAs were instead comparable to that of the other cell lines. Explanations for this discrepancy are provided in the discussion.

### 3.4. *In Situ* Hybridization (ISH) Confirms miR-182 Expression in MCC Cells

**Figure 4:**
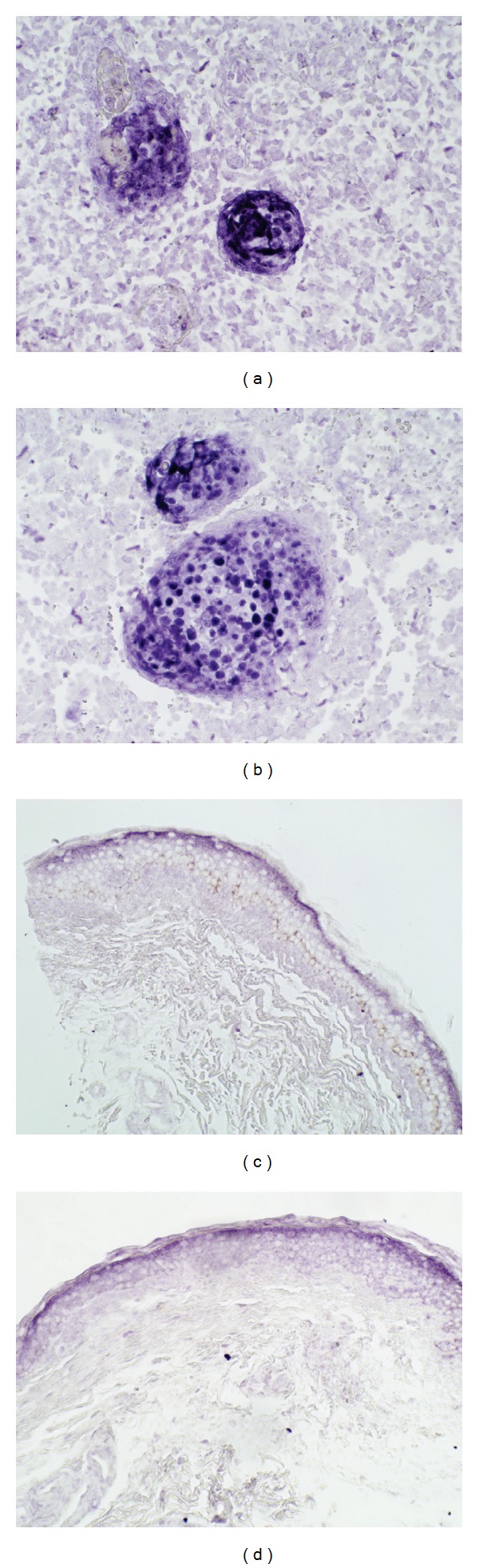
*In situ* hybridization (ISH) confirms that miR-182 is localized to MCC tumor cells. Panels (a) and (b) demonstrate that miR-182 is highly expressed in MCC cells versus the surrounding tissue in a sample of MCC of the cheek; original magnification ×200. Panels (c) and (d) demonstrate that miR-182 expression is low compared to that in MCC cells in a sample of normal skin; original magnification ×200.

To support the notion that these microRNAs play a role in the actual tumor cells in lieu of the surrounding tissue, ISH was performed for one of the more highly expressed MCC-miRs, miR-182, on a sample of MCC of the cheek and on a sample of normal skin (Figure 4). As expected, miR-182 was localized to MCC cells, and, as expected from the qRT-PCR data, expression in surrounding tissue and normal skin was low compared to that in MCC cells.

## 4. Discussion

MCC remains one of the least understood cancers of the skin. MicroRNAs are a relatively young field of biomedical research, born in 2000 with the detection of let-7 in humans, with potential for applications in other pathologies [6, 7]. We believe this will hold true in MCC as well, for which we have identified eight upregulated and three downregulated microRNAs. These MCC-miRs have several implications for the future of MCC research.

### 4.1. MCC-miRs in Cancer: miR-182-183-96

While these MCC-miRs are highly expressed in MCC, some of them have been demonstrated to play significant roles in the pathogenesis of other cancers as well. In particular, the miR-183/96/182 cluster, at chromosomal locus 7q32, is expressed in a diversity of cancers and may contribute to their pathogenesis by targeting multiple components of the cell cycle, DNA damage response, and homologous recombination pathways, and by enriching pathways associated with metastasis, migration, and epithelial-mesenchymal transition [8]. Here we review significant findings in the literature concerning this cluster.

Regarding skin cancer, the miR-183 cluster is frequently overexpressed in melanoma. Our work confirms this observation, inasmuch as miR-182 and miR-183 were upregulated in melanoma versus SCC, BCC, and normal skin (although still not as highly expressed as in MCC). In melanoma, overexpression of miR-182 promotes survival, migration, and metastasis by directly repressing the tumor suppressors FOXO3 and microphthalmia-associated transcription factor-M; and expression of miR-182 increases with progression from primary to metastatic melanoma [9]. This correlation between miR-182 level and aggressiveness is interesting, when noting that MCC, in general, is widely considered to be a tumor with a similar level of aggressiveness as melanoma. It is tempting to entertain the notion that a common pathway may perhaps exist within the two and that a greater dysregulation of said pathway in MCC could account for its high rate of metastasis, morbidity, and mortality. Downregulation of FOX transcription factors is a common theme of this cluster, as miR-183/96/182 have been demonstrated to inhibit FOXO1 in classical Hodgkin lymphoma [10] and in endometrial cancer as well, resulting in decreased G1 cell cycle arrest and cell death [11]. Aberrations of FOX transcription factors have yet to be evaluated in MCC and may be an avenue worth exploring, considering this new information.

The miR-183/96/182 cluster serves essential functions in various noncutaneous carcinomas as well, with much research focused on its role in breast cancer, particularly with invasion and metastasis. For example, in mammary ductal carcinoma *in situ*, both miR-182 and miR-183 have been demonstrated to target CBX7, a regulator of E-cadherin expression [12]. miR-182, activated by *β*-catenin, also targets the matrix metalloproteinase inhibitor RECK, resulting in increased MMP-9 activity [13], as well as MIM, which normally suppresses metastasis by inhibition of RHOA. Dysregulation of these pathways shares the common result of increasing tumor motility and colony formation, and indeed, overexpression of miR-182 in breast cancer cell xenografts results in increased pulmonary colonization by cancer cells [14].

### 4.2. miR-183/96/182: Potential Target Genes Relevant to MCC

The molecular pathways altered in MCC pathogenesis have yet to be fully characterized, but a literature review reveals that some connections to well-known tumorigenic pathways have been made. For example, multiple research groups have discovered that the PI3K/AKT/mTOR pathway is activated, independent of MCPyV-status, in the majority of human MCCs, identifying it as a potential new therapeutic target [15, 16]. Interestingly, the miR-183/96/182 cluster has been demonstrated to enhance PI3K/AKT/mTOR signaling and promote cell migration in medulloblastoma, with the majority of this effect attributed to miR-182. Knockdown of the full cluster in medulloblastoma cells results in dysregulation of the PI3K/AKT/mTOR signaling axis and enhancement of genes related to apoptosis [17]; thus, the miR-183/96/182 cluster again appears as an attractive target for potential therapeutic applications in MCC.

As an additional note, data also suggest that inactivation of PTEN may play a role in MCC pathogenesis; however, mutation and homozygous deletion screening of the PTEN gene in tumor samples reveals nonsense mutations and homozygous deletions in only a small subset of patients [18]. This suggests that alternative mechanisms may exist leading to the inhibition of PTEN. The miR-183/96/182 cluster may provide an explanation to this puzzle, inasmuch as miR-183 has been demonstrated to target the tumor suppressor gene, EGR1, and participate in a miR-183-EGR1-PTEN network that promotes tumorigenesis and cell migration in synovial sarcoma, rhabdomyosarcoma, and colon cancer [19].

### 4.3. Diagnostic Markers: miR-190b, miR-182, and miR-183

Furthermore, some of these MCC-miRs, when employed in combination, could be potentially useful in the diagnosis of MCC. Misdiagnosis is high on the list of issues associated with this cancer; however, unlike BCC, for which it may often be confused [20–22], a delay in diagnosis could prove to be fatal. Cytokeratin (CK) 20 has demonstrated its usefulness in the immunohistochemical (IHC) diagnosis of this cancer; however, there have been reports of CK20−/CK7+ variants of MCC [23, 24]; thus, another tumor marker may aid pathologists. We have demonstrated, via ISH, that miR-182 is indeed localized to the tumor cells (Figure 4).

Recently, Renwick et al. demonstrated that multicolor microRNA FISH can be utilized to effectively differentiate between MCC and BCC in FFPE tissues. The researchers employed miR-205 and miR-375, which were shown to be tumor-specific for BCC and MCC, respectively [25]. Similar methods could be applied with other microRNAs highly expressed in MCC, for example miR-182 and the other identified MCC-miRs in this work, in order to increase the specificity of such a diagnostic test. Although miR-375 was not identified in our study, our MCC-miRs were identified using a larger cohort of MCC samples and have greater versatility as diagnostic markers, inasmuch as they were measured in a wider range of tumors, cell lines, and normal tissues. Thus, they would complement miR-375 well in a multicolor microRNA FISH assay.

In addition, our results from the tissue panel demonstrate that the MCC-miRs could also potentially serve as markers for tumor metastasis (Figure 2). Since baseline levels of these microRNAs are relatively low in target organs such as liver, bone, and brain, among other sites of metastatic involvement, they could potentially serve as markers for MCC cells disseminated in these tissues.

Employing microRNA signatures as diagnostic tools has been successfully carried out for other skin cancers. In 2011, Ralfkiaer et al. developed a qRT-PCR-based classifier consisting of three microRNAs capable of differentiating CTCL from other cutaneous pathologies with high accuracy [26]. As another example, Poliseno et al. developed a microRNA signature that differentiates between superficial and nodular spreading melanoma [27]. These examples demonstrate that microRNA classifiers can potentially function as straightforward disease markers. Perhaps this notion may be practically applied in the context of MCC.

### 4.4. Functional Studies: MS-1

Finally, we demonstrated that several of these MCC-miRs are highly expressed in the patient-derived MCC cell line, MS-1. The same findings were not demonstrated with the MCPyV-negative cell line, MCC13; however, we are not the first to experience such findings. In their previously mentioned study, Renwick et al., upon clustering samples via comparison of microRNA profiles, found that MCPyV-positive cell lines (MS-1, MKL-1, MKL-2) clustered in the MCC group, while MCPyV-negative cell lines (MCC13, MCC26, UISO) clustered in the non-MCC group [25]. Their findings, in addition to ours, support the notion that intrinsic miRNome differences exist between the two cell lines.

This raises the question of which cell line, MS-1 or MCC13, holds more validity as a surrogate of MCC *in vivo*. To address this problem, we refer to the recent work of Guastafierro et al. [28], which characterizes the intrinsic cellular, immunohistochemical, and virological differences between MS-1 and MCC13 in detail. Morphologically, MS-1 and other MCPyV-positive cell lines (MKL-1, MKL-2) grow as floating aggregates in suspension, while MCC13 and other MCPyV-negative cell lines (UISO, MCC26) grow as adherent monolayers in culture. Immunohistochemically, MS-1 is positive for the traditional MCC-markers, CK20 and synaptophysin, and negative for CK7, but in contrast, MCC13 is the exact opposite: negative for CK20 and synaptophysin and positive for CK7. And virologically, MS-1 harbors the integrated viral sequence within its genome and consequently expresses antigens associated with MCPyV-infection and tumorigenesis (e.g., large T antigen), while MCC13 does neither. Taking these differences into account, Guastafierro et al. raise the legitimate question of whether or not the MCPyV-negative cell lines (MCC13, UISO, MCC26, and MaTi) even stem from accurately diagnosed MCC tumors [28].

Based upon these findings, we believe that our results in the MS-1 and MCC13 cell lines corroborate the existing literature that suggests that only the MCPyV-positive cell lines truly mimic MCC. Its cellular, immunohistochemical, and virological features, along with its high expression of MCC-miRs, showcase MS-1 as an attractive candidate for future studies. Further evaluation of the miRNomes of other MCPyV-positive cell lines (MKL-1, MKL-2) would be valuable in supporting this notion.

## Figures and Tables

**Table 1:** MCC sample data. Clinical information corresponding to each of the FFPE MCC samples utilized in qRT-PCR analysis.

#	Age (yrs)	Sex	Race	P/M	Metastasis/invasion/recurrence	Clinical information
1	57	F	W	P	6/9 axillary LNs+, distant LN+	N/A
2	57	F	W	P	Soft tissue involvement, 2/6 LNs+, distant LN+	N/A
3	63	M	W	P	Lumbar spinal cord involvement highly suspected based on FDG-PET/CT imaging	Immunosuppression regimen for renal transplant: mycophenolate mofetil, prednisone, tacrolimus
4	64	M	W	P	N/A	Immunosuppression regimen for renal transplant 2° granulomatosis with polyangiitis: mycophenolate mofetil, prednisone, tacrolimus; history of multiple SCC
5	65	M	W	M	Submandibular gland with soft tissue involvement, 6/24 LNs+, local recurrence	N/A
6	70	M	W	M	Distant (supraclavicular) LN+	History of renal cancer
7	71	M	W	M	Parotid gland with intraparotid LN involvement, local recurrence	N/A
8	72	M	W	P	Local recurrence	History of colon cancer
9	72	M	W	M	Thyroid, parotid gland involvement, 3/23 LNs+	Concurrent papillary thyroid carcinoma
10	76	M	W	P	Distant (cervical) LN+	Concurrent SCC, history of lung cancer
11	76	M	W	P	6/37 axillary LNs+	N/A
12	78	M	W	P	Parotid gland involvement, 9/28 LNs+, local recurrence	N/A
13	79	M	W	P	N/A	History of bladder cancer
14	79	M	W	M	Salivary gland, deep soft tissue surrounding large arteries and skeletal muscle involvement, 14/20 LNs+	N/A
15	80	F	W	M	Multifocal extranodal tumor invasion, soft tissue, and sternocleidomastoid muscle involvement, 11/15 LNs+	History of chronic lymphocytic leukemia
16	82	M	W	P	Parotid gland involvement, distant LN+	History of colon cancer, laryngeal cancer, multiple SCC
17	82	F	W	P	Parotid gland involvement, 2/5 LNs+	History of breast cancer
18	82	M	W	M	Parotid gland with invasion of right upper neck soft tissue, 2/11 LNs+, distant LN+	History of colon cancer, laryngeal cancer, multiple SCC
19	85	M	W	P	N/A	History of acute myeloid leukemia, in remission
20	85	M	W	M	Rectum involvement	History of rectal cancer, prostate cancer

LN: lymph node; M: metastasis; P: primary; W: white.

**Table 2:** MCC-miR candidates identified via NGS. Lists of top fifteen microRNAs upregulated and downregulated in MCC (*n* = 3) versus other tissues (1 melanoma, 1 SCC, 1 BCC, and 1 normal skin sample) and list of fifteen microRNAs expressed in MS-1, based on NGS data.

Upregulated in MCC versus other skin cancers^1^		Upregulated in MCC versus normal skin^1^		Downregulated in MCC versus all other samples^1^		Highly expressed in MS-1	
microRNA	Fold change^2^	microRNA	Fold change^2^	microRNA	Fold change^2^	microRNA	RPKM
miR-885	234.3	miR-183	54.6	miR-455	−100.0	miR-182	441,774
miR-1252	159.4	miR-182	44.3	miR-146a	−33.3	miR-183	406,019
miR-190b	72.3	miR-96	26.4	miR-125b-2	−16.7	miR-10b	368,383
miR-876	69.7	miR-7-2	9.0	miR-224	−16.7	miR-30d	354,071
miR-873	62.2	miR-7-1	8.4	miR-125b-1	−16.7	let-7i	302,976
miR-1468	42.5	miR-769	6.0	miR-452	−12.5	miR-30a	256,918
miR-3065	33.8	miR-708	5.8	miR-27a	−8.3	miR-21	231,060
miR-3074	19.9	miR-93	5.7	miR-503	−6.7	miR-26a	230,375
miR-1250	15.6	miR-106b	5.7	miR-34a	−5.3	miR-9-2	152,761
miR-502	15.2	miR-9-2	4.7	miR-378d-2	−4.8	miR-20a	118,839
miR-660	14.4	miR-532	4.7	miR-24-2	−4.0	miR-532	109,596
miR-501	9.3	miR-9-3	4.7	miR-193a	−4.0	miR-93	108,909
miR-708	9.2	miR-9-1	4.7	miR-378i	−3.6	miR-340	89,562
miR-532	8.2	miR-340	4.0	miR-22	−3.0	miR-7-1	73,329
miR-500a	7.6	miR-192	3.5	miR-34c	−2.9	miR-96a	60,009

^1^Inclusion criteria: >100 reads in all MCC samples; ^2^Fold change (default cut-off ≥ ±2.0) between mapped counts observed between individual groups.
